# Machine learning and synthetic outcome estimation for individualised antimicrobial cessation

**DOI:** 10.3389/fdgth.2022.997219

**Published:** 2022-11-21

**Authors:** William J. Bolton, Timothy M. Rawson, Bernard Hernandez, Richard Wilson, David Antcliffe, Pantelis Georgiou, Alison H. Holmes

**Affiliations:** ^1^ Centre for Antimicrobial Optimisation, Imperial College London, London, United Kingdom; ^2^ AI4Health Centre for Doctoral Training, Imperial College London, London, United Kingdom; ^3^ Department of Computing, Imperial College London, London, United Kingdom; ^4^ National Institute for Health Research, Health Protection Research Unit in Healthcare Associated Infections and Antimicrobial Resistance, Imperial College London, London, United Kingdom; ^5^ Centre for Bio-inspired Technology, Department of Electrical and Electronic Engineering, Imperial College London, London, United Kingdom; ^6^ Department of Critical Care, Imperial College Healthcare NHS Trust, London, United Kingdom; ^7^ Faculty of Medicine, Imperial College London, London, United Kingdom; ^8^ Department of Infectious Diseases, Imperial College London, London, United Kingdom

**Keywords:** antimicrobial resistance, artificial intelligence, clinical decision support systems, decision-making, individualised antimicrobial prescribing, precision prescribing, antibiotic cessation, outcome estimation

## Abstract

The decision on when it is appropriate to stop antimicrobial treatment in an individual patient is complex and under-researched. Ceasing too early can drive treatment failure, while excessive treatment risks adverse events. Under- and over-treatment can promote the development of antimicrobial resistance (AMR). We extracted routinely collected electronic health record data from the MIMIC-IV database for 18,988 patients (22,845 unique stays) who received intravenous antibiotic treatment during an intensive care unit (ICU) admission. A model was developed that utilises a recurrent neural network autoencoder and a synthetic control-based approach to estimate patients’ ICU length of stay (LOS) and mortality outcomes for any given day, under the alternative scenarios of if they were to stop vs. continue antibiotic treatment. Control days where our model should reproduce labels demonstrated minimal difference for both stopping and continuing scenarios indicating estimations are reliable (LOS results of 0.24 and 0.42 days mean delta, 1.93 and 3.76 root mean squared error, respectively). Meanwhile, impact days where we assess the potential effect of the unobserved scenario showed that stopping antibiotic therapy earlier had a statistically significant shorter LOS (mean reduction 2.71 days, p-value <0.01). No impact on mortality was observed. In summary, we have developed a model to reliably estimate patient outcomes under the contrasting scenarios of stopping or continuing antibiotic treatment. Retrospective results are in line with previous clinical studies that demonstrate shorter antibiotic treatment durations are often non-inferior. With additional development into a clinical decision support system, this could be used to support individualised antimicrobial cessation decision-making, reduce the excessive use of antibiotics, and address the problem of AMR.

## Introduction

Bacterial antimicrobial resistance (AMR) is a global threat (1, 2), which resulted in an estimated 1.27 million deaths in 2019 (3). One key strategy to tackle AMR is to optimise antimicrobial use and prolong current antimicrobials’ therapeutic life. Clinical decision support systems (CDSSs) are software designed to provide information to healthcare professionals, patients, or other individuals in order to make informed clinical decisions. With the advent of artificial intelligence (AI) and the ever increasing prevalence of electronic health records (EHRs), numerous CDSSs utilising machine learning (ML) trained on historical patient data have been developed to assist with managing infections (4). Recent research has focused on the diagnoses of bacterial infections (5–7), resistance prediction (8), and antimicrobial therapy selection (9, 10).

One challenge when treating a patient who has a bacterial infection is determining when it is appropriate to stop antibiotic treatment (11). The decision to cease antibiotics too early can result in the patient’s condition worsening, while unnecessary exposure increases the risk of toxicity (12) and drives the evolution of AMR (13). Even over-treating for a short duration can have a significant impact on a population level and enhances the development of resistance (14). Furthermore, excessive treatment is responsible for most avoidable antibiotic adverse events including gastrointestinal distress and allergic reactions (15, 16). Numerous studies have shown that on a population level, shorter treatment durations are often non-inferior to longer ones (17–21). The challenge is that the resulting recommendations do not take into account the individual patient’s characteristics or specific scenarios. It is difficult for clinicians to have confidence in individualised treatment decisions for their patient, when there is a poor understanding of the factors that facilitate or inhibit an individual from receiving a short duration of antibiotic therapy. Therefore, durations are often unnecessarily extended (22) and decided by habit or arbitrarily based on population evidence. Antibiotic cessation should be a collective, data-driven decision, given choices are made in a more favourable environment once time has passed from presentation and significant amounts of information have been gathered. Despite this, systems to help support individualised antibiotic duration and cessation decision-making are often neglected and under-researched with little innovation in this area (23, 24).

**Figure 1:**
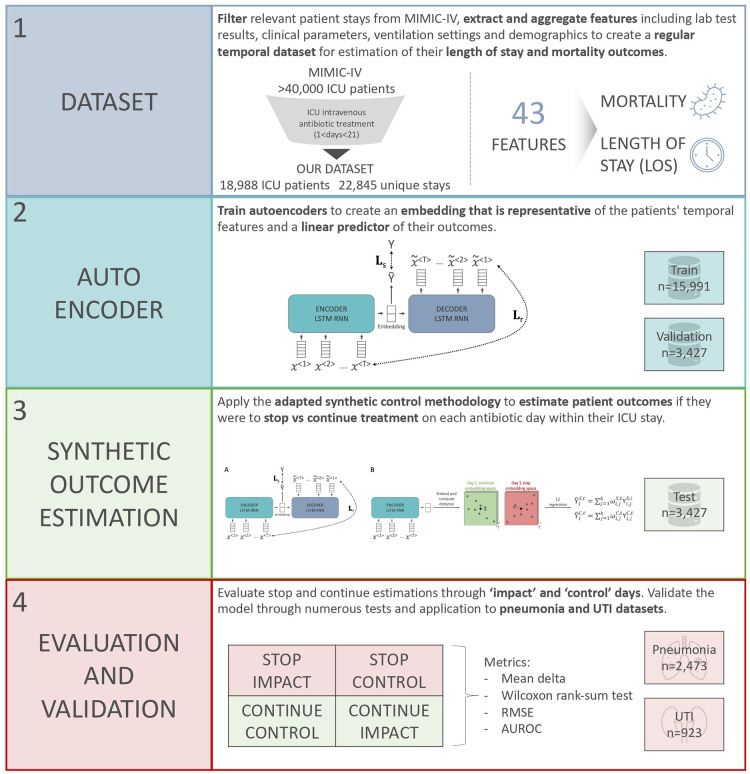
Overview of the steps taken in this research study to develop a model for antimicrobial cessation synthetic outcome estimation.

Given the current standard of care uses clinical factors to determine if a patient should stop antibiotics or not, we hypothesise that an AI-based CDSS using routinely collected EHR data may be able to support individualised antibiotic cessation decision making and overcome prescriber concerns of poor patient outcomes that is likely a major driver of over treatment (25, 26). We approach this problem by estimating clinical outcomes under alternative scenarios with the aim of showing non-inferiority or a direct benefit of antibiotic cessation. More specifically, a machine learning and synthetic control-based approach was developed to estimate patients’ LOS and mortality outcomes for any given day, if they were to stop vs. continue antibiotic treatment. Figure 1 shows a graphical abstract of the approach and methodology employed in this retrospective research study.

## Methods

### Dataset

MIMIC-IV is a large de-identified real-world clinical dataset that is publicly available for clinical research (27, 28). It contains EHR information for over 40,000 patients admitted to the Beth Israel Deaconess Medical Center (BIDMC) in Boston, MA, Unites States, between 2008 and 2019. The patient population was filtered to those who received intravenous antibiotic treatment for a duration between 1 and 21 days during an ICU stay. Input features were extracted, analysed, and selected based on prevalence, correlation, as well as infectious disease doctors and critical care consultants advice. Length of stay (LOS) (continuous value) and mortality (binary) labels were extracted for each patient stay; however, it should be noted that these are not temporally dynamic. An overview of statistics for each dataset is shown in Table 1

**Table 1:** Datasets statistics.

	Dataset					
Statistic	Overall	Train	Validation	Test	Pneumonia	UTI
Number of stays	22,845	15,991	3,427	3,427	2,473	923
Mortality rate	18.60	18.47	18.30	19.52	24.02	18.96
Mean LOS	5.63	5.62	5.74	5.55	9.05	5.50
LOS standard deviation	4.23	4.24	4.31	4.15	5.19	4.32
Mean length of treatment	4.38	4.38	4.48	4.30	6.95	4.77
Length of treatment standard deviation	3.32	3.32	3.48	3.18	4.28	3.55
Spearman’s correlation between LOS and treatment length	0.72	0.72	0.72	0.73	0.73	0.74
Percentage of patients that stopped treatment during their ICU stay	41.56	41.64	41.17	41.55	31.95	26.54

LOS, length of stay; ICU, intensive care unit; UTI, urinary tract infection.

Some features were calculated based on other variables. Cumulative overall antibiotic treatment length was determined for each day of each ICU stay that considered consecutive treatment days irrespective of the antibiotic given. In addition, whether the patient had received re-treatment for antibiotics or not and their age at the time of ICU admission were also computed. Standard pre-processing was applied to features including outliers being removed and values normalised, as well as missing values forward filled or highlighted. Features were aggregated by day for each unique stay to create a regular temporal dataset. In general, there was a high degree of missingness, and so patients with greater than 50% of values missing each day were removed. The resulting dataset contained 43 input features (supplementary Table S1) including lab test results, clinical parameters, ventilation settings, and demographics.

### Model architecture

The objective of our model is to estimate the patients’ LOS and mortality outcomes for any given day, if they were to stop vs. continue antibiotic treatment. It uses a bi-directional long short-term memory (LSTM) autoencoder, which takes in a sequence of patient input features ($x^{\left\langle 1\right\rangle },x^{\left\langle 2\right\rangle }\ldots x^{\left\langle T\right\rangle }$), creates an embedding representation, and outputs a sequence of reconstructed features (${\tilde{x}}^{\left\langle T\right\rangle }\ldots {\tilde{x}}^{\left\langle 2\right\rangle }$, ${\tilde{x}}^{\left\langle 1\right\rangle }$). This autoencoder is trained through two loss functions (29), which are summed together to create a combined loss for backpropagation. First, the reconstruction loss $L_{r}$ is calculated by the root mean squared error (RMSE) between outputs that are trying to reproduce the inputs and the real input data. Second, a supervised learning loss $L_{s}$ is calculated by doing a linear transformation of the embedding representation ($\tilde{Y}$) to try and predict the real label (Y) and taking either the RMSE loss for the LOS outcome or the binary cross-entropy loss for mortality classification. $L_{s}$ ensures that the embedding space created by the autoencoder is a good linear predictor of the outcome of interest, which is important for the subsequent adapted synthetic control method. Overall, an embedding representation is created that considers a patient’s past and is representative of their state on that day.

**Figure 2:**
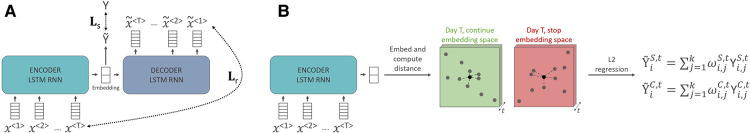
Model illustration. (**A**) The encoder is trained using both a supervised loss (Ls) and reconstruction loss (Lr) (3). (**B**) To estimate outcomes during testing, an embedding is created for every day of each patient’s stay; embedding spaces are partitioned temporally and based on if the patient stopped or continued antibiotics. The closest k neighbours are selected as donors from each embedding space and L2 regression returns weights that minimise the square difference between the patient and the donors. A stop and continue synthetic control outcome is estimated as a weighted average of the donors’ outcomes.

Once the antoencoder is trained and an embedding representation for each antibiotic day in all patient stays have been created, an adapted synthetic control approach (30) is utilised, where the act of stopping or continuing treatment on a particular day is considered an intervention and each patient acts as a singular unit. This method is useful when evaluating an intervention using randomised controlled trials is challenging, as is the case with antibiotic cessation, and hence retrospective observational data are assessed. Synthetic controls have frequently been applied to understand public health interventions (31, 32), but their use within digital health research is limited. In this study, we want to know what are the predicted outcomes if a given patient was to stop vs. continue antibiotics on a given day within their ICU stay. To this extent, two synthetic controls are created, one can be labelled the “stop synthetic control,” which is based on subjects who stopped antibiotics on that particular day, and the second labelled the “continue synthetic control,” which is created from subjects who continue antibiotic treatment on that particular day. To achieve this for each day (t), two separate donor pools are created based on subjects associated embedding representation and antibiotic treatment status. In other words, those who continue antibiotics on day t are partitioned into the “continue” embedding space while those who stop antibiotics are placed in the “stop” embedding space. In this way, the estimated outcomes for stopping and continuing on day t are driven by representative donors who experienced analogous treatment. To create the stop and continue synthetic controls for a particular patient i, the k most closely related to embedding representations from each relevant donor pool are selected based on a distance metric (in this study k=10 and Euclidean distance were used for both stop and continue estimations). Given that embeddings are representative of the patients’ state, those selected donors will be similar, giving a considered insight into potential alternative outcomes under antibiotic temporality. A ridge regression function ($Loss_{i}^{S,t}=\sum_{d=1}^{D}[z_{i,d}^{t}-\sum_{\;j=1}^{k}x_{\;j,d}^{S,t}w_{i,j}^{S,t}{]}^{2}+\sum_{\;j=1}^{k}{w_{i,j}^{S,t}}^{2}$ for stop estimations and $Loss_{i}^{C,t}=\sum_{d=1}^{D}[z_{i,d}^{t}-\sum_{\;j=1}^{k}x_{\;j,d}^{C,t}w_{i,j}^{C,t}{]}^{2}+\sum_{\;j=1}^{k}{w_{i,j}^{C,t}}^{2}$ for continue estimations, where d are the embedding dimensions, j are the donors, and z represents the particular patient $i^{\prime }s$ embedding for a given dimension and time) is then applied to the subject and their respective stop and continue donor embeddings. This returns two sets of weights ($w_{i,j}^{S,t}$ for “stop” and $w_{i,j}^{C,t}$ for “continue”) that minimise the square difference between the subject of interest and the selected units in the donor pools ($Y_{i,j}^{S,t}$ for “stop” and $Y_{i,j}^{C,t}$ for “continue”). The objective of this L2 regularisation is to fairly distribute weights across the donors for stop and continue estimations. Finally, the stop and continue synthetic control outcomes (${\tilde{Y}}_{i}^{S,t}$ and ${\tilde{Y}}_{i}^{C,t}$, respectively) for the particular patient i are computed from the weighted average of donor labels. To this extent during outcome estimation for a given patient i, we assume that we know the outcomes for all other patients within the dataset. Overall outcomes are estimated for each patient on each relevant antibiotic day of their stay if they were to stop vs. continue antibiotic treatment. An overview of the model’s architecture and this process for stop and continue outcome estimation is shown in Figure 2.

### Model development and software

The model was applied on the MIMIC-IV EHR dataset, which was randomly split based on patients’ “stay_id” into training, validation, and testing sets (70%, 15%, and 15%, respectively). PyTorch (33) was used to create a bi-directional LSTM recurrent neural network (RNN) with a custom dataset class to extract labels and features. In order to address the mortality class imbalance (Table 1), over-sampling was used during training. To be specific, those cases with positive mortality were replicated three times within the custom dataset class to achieve a more balanced mortality rate of 51.90% within the train dataset. The Adam optimiser (34) was used with binary cross-entropy loss for classification, mean squared error loss for regression, and Ray Tune for hyperparameter optimisation (35). Training utilised 50 epochs, during which the model with the best performance on the validation dataset (RMSE or area under the receiver operating characteristic curve for LOS and mortality prediction, respectively) was selected as the final model. Two separate LSTM autoencoder models were trained on the whole training dataset to create embedding representations relevant to patients’ LOS and mortality outcomes. Models were evaluated using functions and metrics from the TorchMetrics, Scikit-learn, and SciPy libraries. Further details of the two models’ hyperparameters and their optimisation are shown in the supplementary material (supplementary Figure S1 and Table S2).

### Model evaluation and metrics

**Figure 3:**
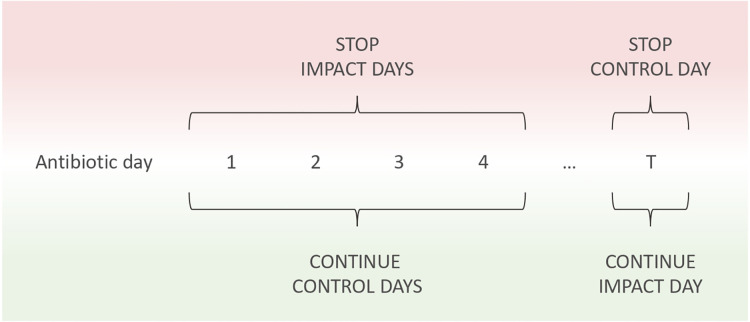
Demonstration of the impact and control evaluation process for stop and continue scenarios. An antibiotic day is defined as each day the patient receives treatment as well as the day they stop. After starting antibiotics, each day the patient receives treatment acts as a stop impact and continue control. This continues until antibiotic cessation or ICU discharge. If the patient stops antibiotics during their ICU stay, that initial day where no antibiotics are administered acts as a stop control and a continue impact.

Commonly with the synthetic control method, the delta difference between the single unit and the counterfactual in the pre-intervention period is minimised and the treatment effect is then observed in the post-intervention period. For our research question, this is not possible due to the nature of stopping antibiotics being the final event at one point in time, after which the patient is not applicable to our research population or question. An analogue can be applied for this study where we define “control” and “impact” days that are equivalent to the pre- and post- intervention periods. For estimating outcomes when continuing antibiotics, all the days the patient actually continues antibiotics are “control” days where we expect minimal difference between the true and estimated outcomes. On the other hand, on the single day the patient stops antibiotics, we can assess the “impact” if they were to instead continue. When estimating outcomes upon stopping antibiotics, the reverse is true, whereby each day antibiotics were continued the “impact” of stopping can be assessed and the final day where the patient stops treatment acts as a “control.” Note that it is not possible to define this for every patient, given not every individual will stop antibiotics during their ICU stay. The percentage of patients who stopped antibiotic treatment during their ICU stay is shown in Table 1. Outcomes are estimated in the same way for impact and control days as discussed in the “Model architecture” subsection. However, for control days, we know the real outcome and so can compare our estimations, while for impact days, the real outcome is unknown. Each day, therefore, acts as both a “control” and “impact” across the two “stop” and “continue” scenario outcome estimations. An outline of this is shown in Figure 3 and the number of continue and stop donors for each day in the test dataset is illustrated in supplementary Figure S2.

For outcome estimation, the mean delta is calculated to evaluate the difference between the real labels and the estimations, through the following formula: $\mu \Delta^{S}=(1/n)\sum_{i=1}^{n}[(1/T_{i})\sum_{t=1}^{T_{i}}[Y_{i}^{S,t}-{\tilde{Y}}_{i}^{S,t}]]$ for stop estimations and $\mu \Delta^{C}=(1/n)\sum_{i=1}^{n}[(1/T_{i})\sum_{t=1}^{T_{i}}[Y_{i}^{C,t}-{\tilde{Y}}_{i}^{C,t}]]$ for continue estimations, where $T_{i}$ is the number of days that the patient receives antibiotics. Minimal difference should be seen on control days where our model aims to reproduce labels, while on impact days you can assess the effect of the unobserved scenario. Statistical analysis can be used to determine if the difference between the true LOS labels and the estimated outcomes are statistically significant. Given the non-normal data distribution, the non-parametric Wilcoxon rank-sum (Mann–Whitney U) test was used with the alpha set at 0.05. Furthermore, the mean absolute percentage error (MAPE) and mean absolute error (MAE) can be calculated through the following notations: ${\mathrm{MAPE}}^{S}=(1/n)\sum_{i=1}^{n}[(1/T_{i})\sum_{t=1}^{T_{i}}\left|Y_{i}^{S,t}-{\tilde{Y}}_{i}^{S,t}\right|/Y_{i}^{S,t}]$ and ${\mathrm{MAE}}^{S}=(1/n)\sum_{i=1}^{n}[(1/T_{i})\sum_{t=1}^{T_{i}}|Y_{i}^{S,t}-{\tilde{Y}}_{i}^{S,t}|]$, respectively, for stop estimations and ${\mathrm{MAPE}}^{C}=(1/n)\sum_{i=1}^{n}[(1/T_{i})\sum_{t=1}^{T_{i}}\left|Y_{i}^{C,t}-{\tilde{Y}}_{i}^{C,t}\right|/Y_{i}^{C,t}]$ and ${\mathrm{MAE}}^{C}=(1/n)\sum_{i=1}^{n}[(1/T_{i})\sum_{t=1}^{T_{i}}|Y_{i}^{C,t}-{\tilde{Y}}_{i}^{C,t}|]$, respectively, for continue estimations. Standard ML metrics can also be used to evaluate model prediction performance. For LOS regression estimation, the RMSE is used, while for the mortality classification task, Area Under the Receiver Operating Characteristic curve (AUROC) is most appropriate given the class imbalance (Table 1), but accuracy, precision, recall, sensitivity, F1 score, and Area Under the Precision Recall curve (AUPRC) can also be calculated. Metrics were calculated as global averages, across all samples, meaning every day of antibiotic treatment within each patients stay is considered equally. 95% confidence intervals were calculated through 1,000 bootstrapped samples on the test set with n=1,000 for mortality metrics and the sum of the squared errors method for LOS RMSE.

To validate our findings beyond the hold out test set, we applied our model to patients who were diagnosed with pneumonia or a urinary tract infection (UTI). The effects of short vs. longer antibiotic treatment regimes have been extensively studied in pneumonia and UTIs. In general, research supports the notion that shorter antibiotic treatments durations are non-inferior to longer ones in these infections, especially for non-complicated cases (19, 36–40). Based on this evidence and the latest antimicrobial prescribing guidelines (41, 42), we defined a long treatment duration as any patient receiving antibiotics for longer than 7 days, and applied our model to estimate their outcomes if they were to instead stop treatment after 7 days. In addition, there is increasing evidence that even shorter courses of antibiotics can be used in such infections, in particular, pneumonia (19, 41). Hence, we investigated the estimated outcomes of those patients who received the standard of care 7 days treatment, for slightly shorter treatment durations (5 or 6 days).

## Results

### Autoencoder

**Figure 4:**
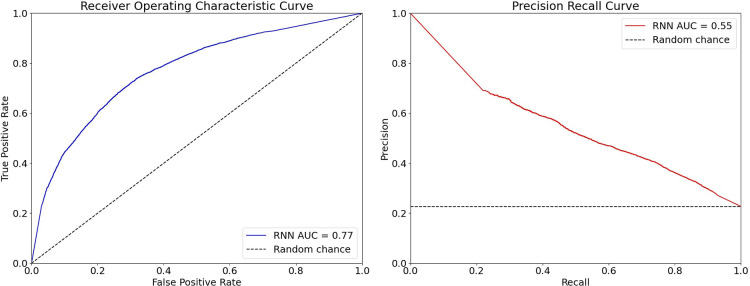
ROC and PRC results for the RNN autoencoder on mortality classification.

In total, 18,988 patients, associated with 22,845 unique ICU stays, were included across datasets. Through a linear transformation of a given patient day embedding, outcome estimations could be made on the unseen test set (3427 unique ICU stays). The LOS model achieved an RMSE of 3.88 (95% CI 3.84–3.92), while the mortality estimation model obtained an AUROC of 0.77 (95% CI 0.73–0.80) [accuracy 0.73 (95% CI 0.71–0.75), precision 0.44 (95% CI 0.36–0.46), recall 0.67 (95% CI 0.61–0.72), specificity 0.75 (95% CI 0.72–0.78), F1 0.53 (95% CI 0.46–0.56), and AUPRC 0.55 (95% CI 0.42–0.56)] (Figure 4), indicating that the model was relatively effective at balancing false-positive and false-negative mortality predictions.

### Synthetic outcome estimation

LOS and mortality estimation results on the unseen test set are shown in Table 2. For LOS estimation on control days, the mean delta under both stopping and continuing scenarios was 0.24 and 0.42 days, respectively, showing a minimal difference between predictions and the ground truth labels. Furthermore, a MAPE of 0.26, MAE of 1.32, and RMSE of 1.93 for stop control days show that the corresponding impact estimations are more reliable. On impact days, stopping earlier had a statistically significant shorter LOS (mean difference 2.71 days, p-value <0.01). This indicates that on average LOS estimations for stopping antibiotics earlier are shorter in duration than those when the patient continues antibiotics. For mortality, no impact was observed by stopping or extending antibiotic treatment. Estimations had modest performance with an average AUROC of 0.67 and accuracy of 0.82; however, the model clearly struggled with false-negative predictions.

**Table 2:** Outcome estimation results for patients in the unseen test set.

		LOS				Mortality		
Scenario	Day(s)	Mean delta (days, p-value)	MAPE	MAE	RMSE	Mean delta	MAE	AUROC
Stop	Impact	2.71*, <0.01	0.36	3.30	4.80	0.06	0.25	0.66
	Control	0.24, 0.60	0.26	1.32	1.93	0.05	0.15	0.72
Continue	Impact	−2.09*, <0.01	0.77	2.85	3.16	0.05	0.18	0.67
	Control	0.42*, 0.01	0.48	2.72	3.76	0.07	0.24	0.64

* Statistical significance with alpha set at 0.05.

LOS, length of stay; MAPE, mean absolute percentage error; MAE, mean absolute error; RMSE, root mean squared error; AUROC, Area Under the Receiver Operating Characteristic curve.

Estimations were made for each day of each patient’s stay within all the extracted data (i.e., train, validation, and testing sets combined) to understand if results would deviate by dataset size. For LOS, reliable estimations were once again obtained (mean stop control difference of 0.33 days and mean continue control difference of 0.42 days). Continuing showed no given impact (mean difference of −0.30 days), while stopping once again showed a significant impact with a mean reduction of 1.87 days. Little difference in mortality estimations was seen between stop and continue controls and impacts (stop impact−0.03, stop control−0.03, continue control−0.05, continue impact−0.05). Mortality predictions were relatively reliable with a mean AUROC of 0.72.

To show the importance of the temporality in our predictions, we created estimations for each antibiotic day of each patients stay, without segregating the embedding space (by time or by antibiotic treatment given they are mutually dependent). The resulting estimations had a mean LOS difference of 2.60 days from the true labels, an RMSE of 5.05, and a statistically significant difference in medians (p-value <0.01).

The performance of the model on subjects towards the edges of the distribution in terms of the correlation between LOS and overall antibiotic treatment length was investigated. Subjects in the 10th and 90th percentiles were selected leading to a smaller Spearman’s correlation of 0.35. As expected, given the dataset size (n=686) and donor distribution, results were quite poor with a mean stop control difference of 2.92 days and a mean continue control difference of 2.13 days. The impact of stopping early though was still much greater than the control at 4.36 days mean difference.

### Pneumonia and UTIs

A total of 2,473 stays where patients were diagnosed with pneumonia were identified, with a mean LOS of 9.05 days and a mean antibiotic treatment length of 6.95 days. Overall estimation of the results on this whole pneumonia population reflected the wider dataset and are shown in Table 3. When focusing on those with long treatment durations and the question of what if they stopped after 7 days of treatment, statistically significant results show that average LOS were 2.82 days shorter when stopping earlier. No difference in estimated mortality was observed; however, estimations were consistent across groups with an average AUROC of 0.75. No significant difference in LOS or mortality was estimated for pneumonia patients who received the standard of care 7 days treatment, if they had slightly shorter treatment durations of 5 or 6 days.

**Table 3:** Outcome estimation results for patients with pneumonia and UTIs.

				LOS		Mortality	
Infection	Analysis	Scenario	Day(s)	Mean delta (days, p-value)	RMSE	Mean delta	AUROC
Pneumonia	Whole dataset	Stop	Impact	3.72*, <0.01	5.87	0.00	0.71
			Control	0.26, 0.47	2.14	0.07	0.76
		Continue	Impact	−2.79*, <0.01	3.65	0.10	0.69
			Control	0.49*, <0.01	4.01	0.05	0.68
	Long treatment durations stopping after 7 days	Stop	Impact	2.82*, <0.01	4.65	−0.03	0.74
			Control	0.43, 0.08	2.11	0.05	0.80
		Continue	Impact	—	—	—	—
			Control	0.41, 0.21	3.47	0.05	0.73
UTI	Whole dataset	Stop	Impact	2.36*, <0.01	4.70	0.14	0.63
			Control	0.36, 0.89	2.04	0.07	0.87
		Continue	Impact	−1.91*, <0.01	3.26	0.03	0.79
			Control	0.38, 0.05	3.82	0.04	0.71
	Long treatment durations stopping after 7 days	Stop	Impact	2.08*, <0.01	4.35	0.30	0.52
			Control	1.04, 0.23	2.42	0.17	0.93
		Continue	Impact	—	—	—	—
			Control	0.26, 0.05	3.48	0.05	0.76

Results are shown for both the whole population and analysis of what if those who received long treatment durations stopped after day 7.

* Statistical significance with alpha set at 0.05.

LOS, length of stay; RMSE, root mean squared error; AUROC, Area Under the Receiver Operating Characteristic curve; UTI, urinary tract infection.

For UTIs, 923 patient stays were selected having a mean LOS and antibiotic treatment length of 5.50 and 4.77 days respectively. Once again, overall estimation results (Table 3) were similar to previous findings with trustworthy controls, stopping early being associated with a shorter LOS and no difference in mortality but reliable estimations (AUROC ranging from 0.63 to 0.87). Estimations for stopping after 7 days for those with long treatment durations did show a positive impact in terms of reduced LOS (mean difference 2.08 days, p-value <0.01). The stop control where we expect to see minimal difference showed a larger mean deviation of 1.04 days, but statistical analysis showed the medians between control estimations and labels were not significantly different. Mortality estimations here were for the most part dependable; a high predictive performance on stop and continue controls was achieved with an AUROC of 0.93 and 0.78, respectively, but a lower score for the stop impact of 0.52. When analysing those patients who received the standard of care 7 days treatment, for slightly shorter treatment durations (5 or 6 days). A statistically significant result was observed where estimated LOS outcomes were on average longer by 1.45 days if the patients stopped antibiotics slightly earlier (p-value <0.01, RMSE 2.72).

## Discussion

**Figure 5:**
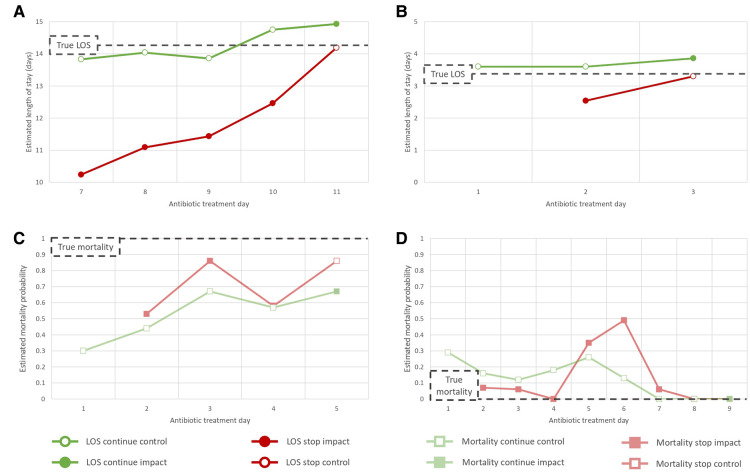
LOS and mortality synthetic outcome estimation results for particular patients. These cases were selected as illustrative examples of four distinct patient scenarios: (**A**) the patient has a long course of antibiotics, (**B**) the patient has short course of antibiotics, (**C**) the patient dies, (**D**) the patient survives. In A/B control estimation results show minimal deviation from the true LOS label while the stop impact estimations have a reduced LOS. Results in C/D indicate mortality estimations are temporally dynamic but with little difference between stop vs. continue.

We demonstrate that our RNN autoencoder and synthetic control-based approach trained on a large ICU EHR dataset can estimate patient outcomes under the alternative scenarios of stopping vs. continuing antibiotic treatment. Results across experiments were consistent, with stop control days often showing the greatest performance indicating our stop impact estimations, which occur on days where the true outcome upon stopping is unknown, are more reliable. The stop impact results from this retrospective study show that stopping antibiotics earlier can be associated with a statistically significant average LOS reduction of 2.71 days. Overall minimal impact on mortality was observed, which is to be expected given death can be caused by a large number of factors beyond those included as model features. Figure 5 shows some specific illustrative examples of patient LOS and mortality estimations. The pneumonia dataset demonstrated particularly positive results with overall and stopping on day 7 analysis indicating antibiotic cessation can have a significant impact on LOS in this population (mean difference 3.72 and 2.82 days, respectively). This reflects current clinical thinking that shorter treatments are optimal for this infection (19, 36, 37, 41). However, there is a balance to be made with antibiotic treatment durations. The UTI analysis indicated courses shorter than 7 days may be detrimental to the patient and that the current standard of care treatment duration is likely appropriate. As such, care must be taken to consider the patients and the public’s best interests with respect to current infections and the threat of AMR.

Our methodological approach to the problem of antibiotic cessation is novel. This model can in principal assist with individualised antibiotic cessation decisions as it takes into account numerous patient characteristics and the specific treatment scenario with regards to patient outcomes, factors that previously could not be considered together in their entirety. This study has approached the problem of antibiotic cessation from the perspective of making a clinically useful tool designed to support decision-making by estimating direct measures that may influence clinical decision-making under alternative scenarios. We believe it could be useful for prescribing physicians during their daily clinical round to compare between stop and continue estimated outcomes and understand when it is appropriate to cease antibiotic treatment. In particular, this system should help show shorter treatment durations can be safe and support individualised antimicrobial decision-making through hard outcome estimation. From a behaviour change perspective, this approach may provide reassurance to support early cessation of therapy, while promoting improved knowledge and understanding on the issue of antimicrobial optimisation and stewardship (43). It should be noted though that too short a course of antibiotics can cause harm and have negative knock-on effects. As such, the aim of this research is to optimise antimicrobial use and determine the most appropriate antibiotic treatment duration for each individual patient. One significant outstanding question is how clinicians treating a patient would adopt recommendations provided by such a system and if it would influence antimicrobial clinical decision-making. Holistically, we believe antibiotic cessation is a collective, data-driven decision, meaning a CDSS in this area can have a larger influence and acceptance by end users. However, the degree to which this tool would be accepted and work alongside clinical decision-making behaviour requires investigation.

We have shown that our model is able to reliably estimate alternative patient outcomes depending on their antibiotic treatment status. Based on our results, the size and consistency of the dataset used and, hence, the number of available donors are strongly related to the reliability of outputs. Experiments utilising small datasets often led to poor results given there were not enough suitable patients within a given embedding space to create an appropriate synthetic estimation. On the other hand, there does seem to be a ceiling above which more instances are not necessary. For example, similar results were obtained across the pneumonia, test, and whole datasets even though they had sizes of 2,476, 3,427, and 22,845 patient stays, respectively. As such, we can infer that this method is likely to produce suitable estimations if several thousand patient examples are available. Although this should be reasonable for most clinical scenarios, it does act as a dataset constraint when evaluating less common infections, where potentially more interesting nuanced findings could be made.

The quality of the initial autoencoder model is another significant implication that determines performance. The standard autoencoder model without the synthetic control methods applied achieved higher performance on the LOS prediction task than estimations generated without segregating the embedding space (RMSE of 3.88 and 5.05, respectively). This confirms first that the model has been trained to appropriately represent the patient in the embedding space with respect to their outcome. Second, the temporal aspect of the embeddings assists with synthetic outcome estimations and finally the subsequent synthetic outcome estimation methodology applied ensures that outputs can be clinically applicable with regards to antibiotic treatment. As such, the autoencoder is critical for appropriate temporal representations and subsequent estimations.

It is important to note that there is a high degree of correlation between LOS and overall treatment length in the datasets (Table 1, supplementary Figure S3). This is to be expected given those patients who are less sick will likely receive fewer antibiotics and leave the ICU sooner. Although the model architecture is designed to account for this, through representative and segregated embeddings, it is still likely that the model “learned” this association causing some confounding. Results on outliers when there is reduced correlation still illustrate that stopping can impact LOS outcomes, even if the predictions themselves are not reliable in this situation given the skewed dataset analysed. Numerous factors influence ICU LOS; hence, even if the model predicts that stopping antibiotics could be neutral or beneficial, other random factors may make this an impossibility. Nevertheless, our results and the strong correlation observed between antibiotic treatment length and LOS in this dataset mean this model can act as a proxy with the ultimate aim of reducing the unnecessary use of antibiotics.

This study has several limitations. We focused on addressing what would happen if antibiotic cessation occurred earlier during a patient’s ICU stay. The synthetic control methodology was chosen and adapted as it allows us to address this problem while more traditional causal discovery seems intractable. MAPE and MAE LOS estimation results are in the region of days which could limit clinical utility but are comparable to that of recent research (44). Unlike most synthetic control applications, we do not have an extensive pre-intervention period making confidence in results more challenging. Furthermore, one of our analogues stop “control” days would not be available on a patient-specific level during clinical use due to the nature of cessation occurring after treatment. Other types of interpretability such as being able to investigate selected donors to see if they are clinically meaningful could counteract this. Second, the use of historical EHR data to estimate the synthetic outcome means all our estimations are biased based on past antibiotic prescribing policies. These methodological approaches were required to answer our question of interest but mean that historical approaches towards antimicrobial stewardship govern our model’s outputs. The analysis of such a large dataset along with estimations being the weighted average of donors does, however, mitigate this to some extent. In conjunction with this, the analysis presented here is of a macro-scale; however, to realise the potential of this approach for true antimicrobial optimisation, more nuanced, relative, and individualised studies will be required, which we plan to conduct in future. Finally, given the high degree of missingness in the dataset, a number of clinically important features have to be excluded. In particular, research shows that procalcitonin (PCT) and C-reactive protein (CRP) are useful biomarkers for determining when it is safe and appropriate to stop antibiotic therapy (45–48). Neither of these were included as features due to insufficient data. As such, this approach and the subsequent results could potentially be more powerful if applied to a complete dataset focused on a narrow type of infection with defined variables of interest.

In conclusion, we have developed an AI-driven model to estimate patient outcomes if they were to stop or continue antibiotic treatment in the ICU. With further development into a CDSS, we envisage that this can assist clinicians with antimicrobial optimisation and reduce the excessive use of antibiotics to tackle AMR. Future research will investigate which variables promote or hinder cessation and discern the ability of this tool to influence antimicrobial decision-making.

## Data Availability

Publicly available datasets were analysed in this study. These data can be found here: https://physionet.org/content/mimiciv/1.0/.
