# Supplementary material for: Machine learning and synthetic outcome estimation for individualised antimicrobial cessation
Source: Front Digit Health. 2022 Nov 21;4:997219. doi: 10.3389/fdgth.2022.997219 (PMC9719971; doi:10.3389/fdgth.2022.997219)
Supplement: Supplementary file 1 [file Datasheet1.pdf]

Supplementary Material

1 SUPPLEMENTARY TABLES AND FIGURES

1.1 Figures

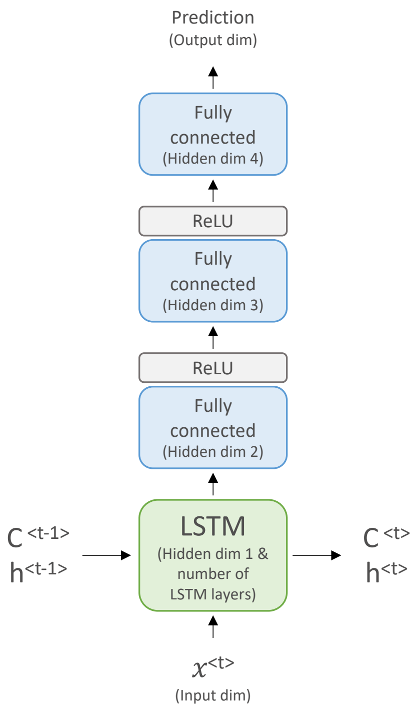

Figure S1. Overview of an LSTM cell within the autoencoder model for any given time-step. Dimension sizes are shown in S2. Note a final sigmoid activation function (not shown) is applied to the output for mortality prediction.

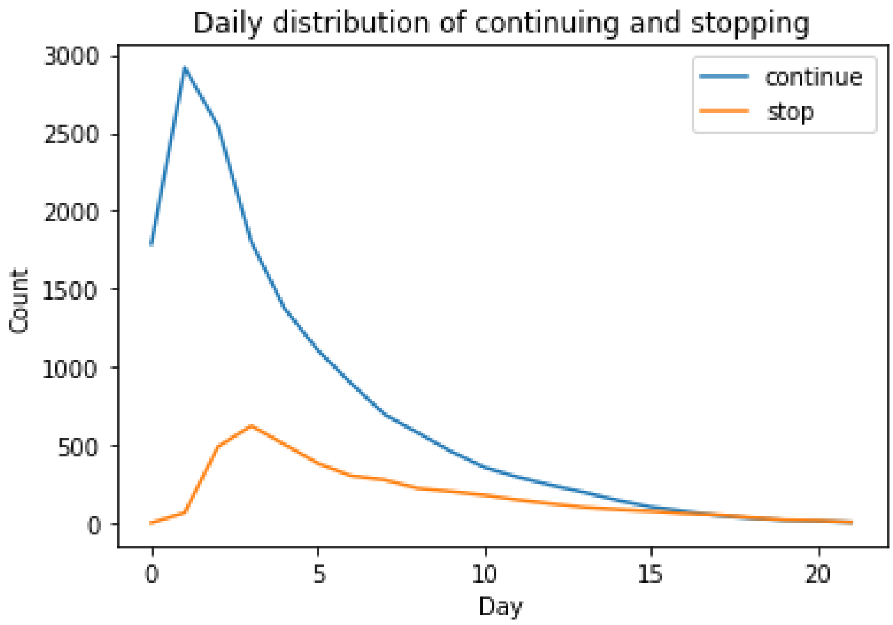

Figure S2. Distribution of continuing and stopping antibiotic treatment by day within the test dataset. Excluding the particular patient i whose outcomes are being investigated, those who continue on a given day are within the continue donor pool, while those that stop on that day are within the stop donor pool.

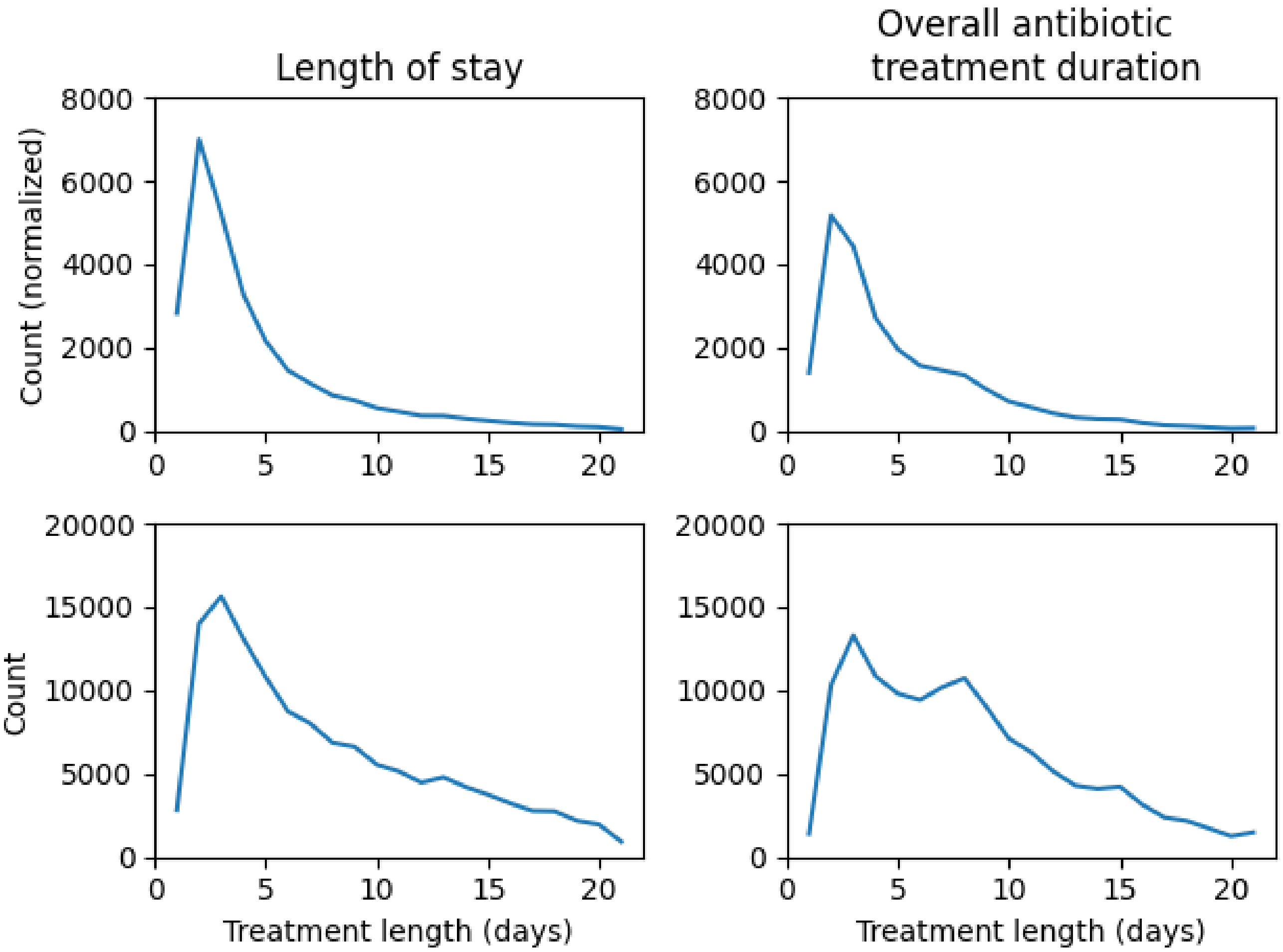

Figure S3. Overall antibiotic treatment duration and length of stay for patients. The normalised count represents the number of distinct occurrences for that specific day, while the raw count represents the number of days of data available.

1.2 Tables

Table S1. Autoencoder input features.

Input variable	Degree of missingness (%)
Glasgow Coma Score - Eye Opening	63.4
Glasgow Coma Score - Verbal Response	63.4
Glasgow Coma Score - Motor Response	63.4
Heart Rate	55.3
Respiratory Rate	55.6
O2 saturation pulseoxymetry	55.9
Temperature	59.3
Foley	34.0
Base Excess	54.9
Lactate	63.7
pCO2	55.0
pH	50.1
pO2	55.6
Alanine Aminotransferase (ALT) 0	66.6
Alkaline Phosphatase (ALP)	64.9
Asparate Aminotransferase (AST)	66.0
Anion Gap	5.8
Bicarbonate	5.0
Bilirubin	66.7
Calcium	10.7
Chloride	4.8
Creatinine	11.3
Glucose	8.6
Magnesium	8.8
Phosphate	12.2
Potassium	5.3
Sodium	5.6
Urea Nitrogen	7.5
Hemoglobin	4.7
Mean corpuscular hemoglobin (MCH)	6.2
Mean cell hemoglobin concentration (MCHC)	4.5
Mean corpuscular volume (MCV)	5.6
Platelet Count	6.2
Prothrombin time (PT)	36.1
Partial thromboplastin time (PTT)	36.4
Red cell distribution width (RDW)	6.1
White Blood Cells	6.8
Cumulative treatment length	0
Re-treatment	0
Insurance	0
Ethnicity	0
Sex	0
Age	0

Table S2. Model hyperparameters.

Hyperparameter	LOS model	Mortality model	Optimisation options
Learning rate	0.0001	0.00001	0.00001, 0.0001, 0.001, 0.01
Batch size	512	512	32, 64, 128, 256, 512
Input dimension	43	43	-
Hidden dimension 1	128	128	-
Hidden dimension 2	512	256	16, 32, 64, 128, 256, 512
Hidden dimension 3	256	128	16, 32, 64, 128, 256, 512
Hidden dimension 4	2	2	-
Output dimension	1	1	-
Number of LSTM layers	5	2	1, 2, 3, 4, 5
Bidirectional encoder	True	True	True, False
Activation function	ReLU	ReLU	-
